# Detection of *Leptospira* spp. in Water Turtle (*Trachemys scripta*) Living in Ponds of Urban Parks

**DOI:** 10.3390/vetsci4040051

**Published:** 2017-10-10

**Authors:** Daniela Dezzutto, Raffaella Barbero, Giuseppina Canale, Pier Luigi Acutis, Cristina Biolatti, Andrea Dogliero, Mauthe Degerfeld Mitzy, Paola Francone, Alberto Colzani, Stefania Bergagna, Maria Silvia Gennero

**Affiliations:** 1 Istituto Zooprofilattico Sperimentale del Piemonte, Liguria e Valle d’Aosta, 10154 Torino, Italy; raffaella.barbero@izsto.it (R.B.); giuseppina.canale@izsto.it (G.C.); pierluigi.acutis@izsto.it (P.L.A.); cristina.biolatti@izsto.it (C.B.); stefania.bergagna@izsto.it (S.B.); mariasilvia.gennero@izsto.it (M.S.G.); 2 Dipartimento di Scienze Veterinarie, 10095 Grugliasco, Italy; andrea.dogliero@unito.it (A.D.); mitzy.mauthe@unito.it (M.D.M.); 3 Azienda sanitaria Locale TO1—Servizio Veterinario, 10128 Torino, Italy; paola.francone@aslto1.it (P.F.); alberto.colzani@aslto1.it (A.C.)

**Keywords:** *Leptospira* spp., water turtle, urban parks

## Abstract

Urban parks are green areas of cities where families and children spend hours outside. Turtles often inhabit urban parks. However, even if the animals seem harmless, they may serve as both reservoirs or accidental hosts for different serotypes of *Leptospira* spp. *Leptospira* spp. is a waterborne zoonotic bacterium relevant for public health. Reptiles and amphibians may play a role in the epidemiology, transmission, and persistence of *Leptospira* spp. In the present study, we observed the presence of anti-leptospiral agglutinins in a group of freshwater turtles (*Trachemys scripta*) captured in three urban ponds of the metropolitan city of Turin, Italy.

## 1. Introduction

Leptospirosis is one of the most important zoonotic diseases. The etiologic agent is a pathogenic bacteria of the genus *Leptospira* that may cause high mortality and morbidity. The main *Leptospira* reservoirs are rodents, but different species can function as maintenance hosts. *Leptospira* spp. is a waterborne zoonotic bacterium relevant for public health. Many wild and domestic mammals act as accidental hosts for various serotypes of *Leptospira* spp.

However, some herpetofauna—particularly reptiles and amphibians—may play a reservoir role when considering epidemiology, transmission, and persistence of *Leptospira* spp.

In recent years, an increase of positive dogs to the serological test for *Leptospira* was recorded in the Piedmont Region, and for two consecutive years the Turin Public dog house has been closed following an outbreak of leptospirosis. The most relevant serogroups were *L. bratislava*, *L. copenagheni*, *L. tarassovi*, *L. hardjo*, *L. icterohaemorragiae*, *L. canicola*, and *L. pomona*.

In just the two last years, 437 serological tests have been performed at our laboratories for the search for *Leptospira* in dogs, and 87 were positive. This rise in cases has led researchers to look for some possible causes.

Infection by *Leptospira* spp. in humans occurs through direct contact with infected animals or indirectly by contact with a contaminated environment [1]. The main causes of transmission to humans are contaminated water or direct contact with abortion fluids or the infected urine of rodents and livestock animals [2]. The identification and characterization of new bacterial strains have increased our understanding of the epidemiology of the various leptospiral strains [3]. However, the *Leptospira* genus presents a different taxonomic classification between the bacterial phyla, with both its serological and genetic features useful for phylogenetic identification [4]. Taxonomic classification includes more than 300 serovars divided into 24 serogroups and several species, and includes genomic features and serological analyses. *Leptospira* spp. is distributed throughout the world, and its occurrence is advanced in tropical areas—especially during seasonal rains [5]. Leptospiral strains are broadly distributed in soil and water worldwide. As described by Bharti et al., the group of natural reservoirs of pathogenic leptospires is vast and continues to increase [6,7,8].

In the present study, we observed the presence of anti-leptospiral agglutinins in a group of freshwater turtles (*Trachemys scripta*) captured in three urban ponds of the metropolitan city of Turin, Italy. The serological method used for diagnosis was the microscopic agglutination testing (MAT).

## 2. Materials and Methods

### 2.1. Animal Selection and Sampling

The present study was conducted in three urban ponds in Turin, Piedmont Region (Northwest Italy). These ponds were selected to determine the health risks associated with turtles present in the parks, as it is estimated that more than one thousand turtles can be present .The reason why so many turtles are present in an urban park is that they are often abandoned by private citizens. Sixteen *Trachemys scripta* (14 *Trachemys scripta scripta* and 2 *Trachemys scripta elegans*) were captured using net poles. Each animal was subjected to a careful medical examination by veterinarians to assess the health status of the 16 subjects.

Blood samples were collected from the cervical vertebral venous plexus and taken into tubes without anticoagulants. Serum samples were separated by centrifugation at 3500× *g* for 5 min at room temperature, and analyzed visually for hemolysis that could affect the results of the analyzes. Serum obtained was stored at −20 °C until analysis.

### 2.2. Serology

Serological analysis was performed according to the recommendation of the World Organization for Animal Health (OIE), using microscopic agglutination test (MAT) with antigens, and a microscope equipped with a dark field condenser, as previously described. These antigens were maintained in liquid media (EMJH), as recommended by OIE [9].

The test currently used as gold standard for leptospirosis detection in humans and animals is the microscopic agglutination test. However, more than 250 serovars have been described to-date, and therefore use of the MAT requires the most geographically relevant panel of serovars.

The procedure suggested by Cole et al. [10] was realized in a round-bottom 96-well microtiter plate. A volume of 50 µL of sterile PBS (pH 7.4) was introduced to each well. Serum samples were diluted using separate tubes (1:25) and added to the well to make a 1:50 dilution. For each well, the concentration of the organism in dark field microscopy was checked and a constant amount of 50 µL of each antigen was added. After the addition of the antigen, the final dilution varied from 1:100 to 1:6400. In addition, positive and negative controls were included in the experiment. The plates obtained were incubated at room temperature (37 °C) for 2 h. At the end of the incubation, part of the samples were taken on a clean glass plate from the maximum dilution and examined in dark field microscopy. The end point of an agglutination reaction was considered to be the highest dilution in which 50% of the leptospires were agglutinated. The reciprocal of the final point formed the titer [11]. In the present study, the titer 1:100 was considered as positive for MAT.

At the end of the incubation, a loopful of sample was taken on a clean glass slide beginning from the highest dilution and examined under dark field microscopy (DFM) without cover glass. The end point of an agglutination reaction was taken as the highest dilution at which 50% of leptospires had agglutinated. The reciprocal of the end point formed the titer. A titer of 1:100 was considered a positive titer by MAT [11].

As recommended, the serum samples were initially tested using a 1:50 dilution, and those with agglutination level equal to or greater than 50% were further diluted [12]. The final titration was determined as the dilution where the level of agglutination of 50% was observed. All reactions were analyzed by the same technician to avoid biased interpretation. Titration of 1:50 was used to indicate that the animals were exposed to the etiologic agent. Titration ≥1:100 were considered positive for *Leptospira* infection. 

The panel of antigens used in this study was composed by ten standard strains considered the most prevalent, with the following serogroups: *L. ballum*, *L. bataviae*, *L. bratislava*, *L. copenagheni*, *L. tarassovi*, *L. hardjo*, *L. icterohaemorragiae*, *L. canicola*, *L. pomona*, *L. saxkoebing*.

## 3. Results and Discussion

Table 1 shows the obtained results. Leptospirosis is a zoonotic infectious disease affecting animals all over the world. The gold standard for the serological diagnosis of leptospirosis is the microscopic agglutination test.

Although there is a new genospecies classification of the genus *Leptospira*, this test is a valid reference for the diagnosis of the disease and the test due to its proven efficacy. The microscopic agglutination test allows identification of the agglutinating antibodies against the external membrane lipopolysaccharide (LPS) shared by *Leptospira* spp. The presence of wild animals in human-dominated landscapes allow overlap between humans, wildlife, and domestic animals. 

This cohabitation represents a health risk for sensitive animals, including humans exposed during leisure activities and professionals working with freshwater turtles [12].

In the current study, only four turtles were negative for MAT to all serovars tested. Among positive animals, the most common serovar identified was *L. tarassovi*, with a frequency of 62.5%. The serum antibody titers ranged between ≥1:50 and ≤1:3200. The second most common serovar identified in the current study was *L. saxkoebing*, with 31.25%. *L. copenagheni* and *L. canicola* serovars were identified in a single turtle. We observed lower titers of antibodies reactive (≤1:200) to *L. saxkoebing*, *L. copenagheni*, and *L. canicola*. All other serovars were negative to MAT.

This investigation has allowed us to observe the presence of anti-leptospiral agglutinins in freshwater turtles and to confirm the exposure of these animals to *Leptospira*. 

It is not possible to know the real risks related to the presence of turtles in the city lakes for other animal species cohabiting urban areas for both children playing in parks. To confirm this risk, however, further studies are needed to isolate the spirochete in seropositive turtles or in the waters of the lakes. 

Official Veterinarians (ASL TO1) and the Città Metropolitana di Torino have taken steps to limit direct contact with animals. Furthermore, an information campaign was launched to prevent infection with *Leptospira* spp.

This work is a demonstration of how a survey can be useful to prevent disease without having to take drastic action with regard to animals. Disease prevention and risk communication are the basis for a good coexistence between humans and animals.

## Figures and Tables

**Table 1:** The obtained results with microscopic agglutination test (MAT) used to detect leptospirosis in turtles in three different lakes FL: free pond; SL: small pond; LL: large pond.

Sample	Pond	*L. tarassovi*	*L. copen-hageni*	*L. cani-cola*	*L. saxkoe-bing*	*L. ballum*	*L. bataviae*	*L. bratisl-ava*	*L. hardio*	*L. icterohaem-orragiae*	*L. pomona*
*Turtle 1*	FL	-	-	-	-	-	-	-	-	-	-
*Turtle 2*	FL	1:400	1:100	1:50	-	-	-	-	-	-	-
*Turtle 3*	FL	1:100	-	-	-	-	-	-	-	-	-
*Turtle 4*	FL	1:50	-	-	-	-	-	-	-	-	-
*Turtle 5*	FL	-	-	-	-	-	-	-	-	-	-
*Turtle 6*	FL	1:400	-	-	-	-	-	-	-	-	-
*Turtle 7*	FL	1:50	-	-	1:200	-	-	-	-	-	-
*Turtle 8*	SL	1:100	-	-	-	-	-	-	-	-	-
*Turtle 9*	SL	1:3200	-	-	-	-	-	-	-	-	-
*Turtle 10*	SL	-	-	-	1:100	-	-	-	-	-	-
*Turtle 11*	SL	-	-	-	1:50	-	-	-	-	-	-
*Turtle 12*	LL	-	-	-	-	-	-	-	-	-	-
*Turtle 13*	LL	1:50	-	-	-	-	-	-	-	-	-
*Turtle 14*	LL	1:200	-	-	1:50	-	-	-	-	-	-
*Turtle 15*	LL	1:3200	-	-	1:200	-	-	-	-	-	-
*Turtle 16*	LL	-	-	-	-	-	-	-	-	-	-
